# ARE PERCEIVED NEIGHBORHOOD CHARACTERISTICS ASSOCIATED WITH CHRONIC PAIN IN MIDDLE-AGED AND OLDER ADULTS?

**DOI:** 10.1093/geroni/igac059.1937

**Published:** 2022-12-20

**Authors:** Yulin Yang

**Affiliations:** University of California, San Francisco, San Francisco, California, United States

## Abstract

Neighborhoods predict many health outcomes including functional limitations and disability; however, how neighborhoods are associated with chronic pain remains poorly understood. This study uses examined the association between four perceived neighborhood characteristics (social cohesion, physical disorder, safety, and social ties) and chronic pain in a nationally representative sample of adults aged 51+ in the US. Among 14,069 participants (58.65% women; mean age 68.81) in the Health and Retirement Study (2006-2008), we investigate whether neighborhood social cohesion, neighborhood physical disorder, neighborhood safety, and neighborhood social ties were associated with odds of having moderate-severe and limiting pain using adjusted logistic regression. After adjusting socio-demographic factors (age, gender, race/ethnicity, education, wealth, marital status), residence in neighborhoods with high perceived social cohesion, safety, and more social ties were associated with lower odds of having moderate-severe and limiting pain. Perceived neighborhood physical disorder did not predict whether respondents have moderate-severe and limiting pain or not.

